# miR-33a is up-regulated in chemoresistant osteosarcoma and promotes osteosarcoma cell resistance to cisplatin by down-regulating TWIST

**DOI:** 10.1186/1756-9966-33-12

**Published:** 2014-01-27

**Authors:** Yong Zhou, Zufa Huang, Song Wu, Xiaofang Zang, Min Liu, Jian Shi

**Affiliations:** 1 Department of Orthopaedics, The Third Xiangya Hospital, Central South University, 138 Tongzipo Road, Changsha, Hunan 410013, China

**Keywords:** microRNA, miR-33a, TWIST, Osteosarcoma, Chemoresistance, Apoptosis

## Abstract

**Background:**

miRNAs are involved in osteosarcoma (OS) chemoresistance, and TWIST reportedly enhances cisplatin-induced OS cell apoptosis by inhibiting multiple signaling pathways. In this study, we profiled miRNAs differentially expressed in chemoresistant OS, with a focus to identify miRNAs that regulate TWIST expression and OS chemoresistance.

**Methods:**

OS patients who showed <90% tumor necrosis after neochemotherapy were defined as poor responders (chemoresistant), and those who showed ≥90% tumor necrosis were defined as good responders (control). miRNA microarray analysis was carried out with a discovery cohort (n = 12) of age-, sex- and tumor stage-matched chemoresistant and control OS patients.

**Results:**

Among the up-regulated miRNAs in chemoresistant OS samples, miR-33a was verified to down-regulate TWIST expression, which was supported by an inverse miRNA-33a/TWIST expression trend in the validation cohort (n = 70), target-sequence-specific inhibition of TWIST-3′ untranslated region-luciferase reporter activity by miR-33a, and alteration of TWIST expression by overexpression or inhibition of miR-33a in human OS cell lines. In Saos-2 cells treated with cisplatin, inhibition of miR-33a by antagomir-33a markedly increased cell apoptosis, which was enhanced by overexpression of TWIST. The apoptosis-inducing effect of TWIST overexpression was reversed by overexpression of miR-33a. In MG-63 cells, overexpression of miR-33a significantly decreased cisplatin-induced cell apoptosis, which was enhanced by knockdown of TWIST. Antagomir-33a significantly increased cisplatin-induced cell apoptosis, which was reversed by knockdown of TWIST.

**Conclusions:**

We have demonstrated in this study that miR-33a is up-regulated in chemoresistant OS and that the miR-33a level is negatively correlated with the TWIST protein level in OS. Our in vitro data indicate that miR-33a promotes OS cell resistance to cisplatin by down-regulating TWIST; on the other hand, inhibition of miR-33a by antagomir-33a enhances cisplatin-induced apoptosis in OS cells by up-regulating TWIST expression. The findings suggest that inhibition of miR-33a/TWIST signaling could be a potential new strategy to enhance neoadjuvant chemotherapy for OS.

## Background

Osteosarcoma (OS) is the most frequent malignant bone tumor in children and adolescents, comprising 2.4% of all malignancies in pediatric patients [1]. The 5-year survival rate of OS patients has significantly improved over the past decades to approximately 60-70% since the introduction of combinatorial chemotherapy [2]. However, a significant proportion of OS patients still respond poorly to chemotherapy, and they have a risk of local relapse or distant metastasis even after curative resection of the primary tumor and intensive chemotherapy. Standard chemotherapy of OS is based on a combination of different drugs: neoadjuvant therapy with methotrexate, cisplatin, and doxorubicin followed by surgery and post-operative chemotherapy (methotrexate, cisplatin, doxorubicin, cyclophosphamide, and vincristine). Despite this, approximately 30% of patients relapse or develop metastasis [3]. The lack of responsiveness to chemotherapy due to intrinsic or acquired chemoresistance is the major reason for poor survival and disease relapse of OS patients [4]. Recently, novel molecular targeted drugs have emerged, but they have not been well established for the treatment of OS [5]. In addition, the molecular mechanisms underlying OS chemoresistance remain largely obscure. Hence, identification of factors that contribute to OS chemoresistance and elucidation of the underlying mechanisms will be pivotal in the development of new therapeutic strategies.

TWIST, also known as TWIST1, belongs to the basic helix-loop-helix (bHLH) transcription factor family. During embryonic development, TWIST plays an essential role in specification of the mesoderm and differentiation of the mesoderm-derived tissues [6]. Twist haploinsufficiency was shown to upset bone tissue in both mice and humans [7,8]. In homogeneous cohort of OS patients, the *TWIST* gene was frequently deleted in the tumors at diagnosis, and its haploinsufficiency was significantly correlated with a poorer patient outcome [6,9]. It has been reported that TWIST decreases OS cell survival against cisplatin by inhibiting β-catenin signaling and endothelin-1/endothelin A receptor signaling pathways [10,11], suggesting that TWIST is an important negative regulator in the development of OS chemoresistance.

MicroRNAs (miRNAs) are noncoding small RNAs, usually 18–25 nucleotides in length, which repress translation and cleave mRNA by base-pairing to the 3′-untranslated region (UTR) of the target genes [12]. Knowledge of individual miRNAs effecting developmental biology, cellular differentiation programs, and oncogenesis continues to grow [13]. Differences in the miRNA expression profiles detected between cancer cells and their normal counterparts have revealed that miRNAs are involved in the pathogenesis of cancer [14]. In addition, miRNAs may play multiple roles as tumor suppressors, oncogenes, or both in some cases [15]. The biological properties of miRNAs may make them useful as diagnostic and prognostic tools as well as therapeutic targets in various cancers, including OS. A number of miRNAs reportedly are involved in OS tumorigenesis and chemoresistance [13].

In the present study, we screened for miRNAs regulating TWIST expression in human OS and explored their functional interaction in modulating human OS chemoresistance.

## Methods

### Patients

From November 2010 to May 2013, we enrolled two cohorts of OS patients. The discovery cohort consists of six Han Chinese OS patients who showed <90% tumor necrosis (mean 70.8% ± 9.2%) after chemotherapy and were defined as poor responders at the third Xiangya Hospital of Central South University [16]. Another six age-, sex-, and tumor stage-matched Han Chinese OS patients, who showed ≥90% tumor necrosis (mean 94.1% ± 2.8%) as good responders [16], were enrolled as controls. In the validation cohort, 35 Han Chinese poor responders and 35 Han Chinese good responders were enrolled. All patients had OS in the long tubular bones and were treated preoperatively with neoadjuvant chemotherapy as follows: intravenous (i.v.) doxorubicin (3 courses at 25–30 mg/m^2^/day for 3 days), i.v. methotrexate (4 courses of up to 14 g/m^2^/day for 1 day) and intra-arterial cisplatin (3 courses at 35 mg/m^2^/day for 3 days). All OS diagnoses were based on biopsy and the response to treatment was determined histologically as the percentage of necrosis after neoadjuvant chemotherapy. Patients with any other malignancies or a family history of OS or any other cancers were excluded. Baseline characteristics of all 82 patients are summarized in Table 1. This study was approved by the Ethics Committee of the Third Xiangya Hospital, Central South University. Written informed consent was obtained from the parent or guardian of minor participants before the start of the study.

**Table 1:** Characteristics of study cohorts

**Group**	**Discovery cohort**		**Validation cohort**	
	**Chemoresistant OS (n=6)**	**Control (n=6)**	**Chemoresistant OS (n=35)**	**Control (n=35)**
Age (years)				
Mean±SD	9.6±4.2	9.6±4.2	9.9±4.9	10.1±5.2
Range	7–14	7–14	6–16	6–16
Gender n (%)				
Male	3 (50)	3 (50)	21 (60)	23 (66)
Female	3 (50)	3 (50)	14 (40)	12 (34)
Tumor stage n (%)				
Stage IIA	2 (33)	2 (33)	8 (23)	7 (20)
Stage IIB	2 (33)	2 (33)	13 (37)	13 (37)
Stage III	2 (33)	2 (33)	14 (40)	15 (43)
Body mass index (kg/m^2^)	17.5±4.0	18.2±4.1	17.7±3.8	18.3±4.0
Tumor necrosis (%)	94.1±2.8	70.8±9.2*	93.5±3.6	73.6±12.2*

Note: *OS* osteosarcoma. *p < 0.05 vs Chemoresistant OS.

### Cells lines, reagents and plasmid constructs

Saos-2 and MG-63 human OS cell lines were purchased from the American Type Culture Collection (Manassas, VA, USA). Human Twist cDNA was subcloned into the pcDNA 3.1 expression vector [17]. Twist (sc-38604-V) short hairpin RNA (shRNA) lentiviral particles, control shRNA lentiviral particles-A (sc-108080), and anti-TWIST (sc-81417) antibody were purchased from Santa Cruz Biotechnology (Santa Cruz, CA, USA). The DeadEnd™ Fluorometric TUNEL System was purchased from Promega (Madison, WI, USA). Superfect™ transfection reagent was purchased from Qiagen (Valencia, CA, USA). Dual-luciferase reporter assay system was purchased from Promega (Madison, WI, USA). Puromycin, cisplatin, and all chemicals of reagent grade were purchased from Sigma (St. Louis, MO, USA). The 3′-UTR of TWIST was amplified from genomic DNA using the following primers: 5′-GCGCCTCGAGCAGGCGGAGCCCCCCACCCCCTCA-3′ (forward) and 5′-GCGCGCGGCCGCGCAGAAAAATATACAAAGATATT-3′ (reverse). The TWIST-3′UTR-luciferase reporter was generated by inserting the TWIST 3′-UTR between XhoI and NotI restriction sites (underlined in the above primers) of the psiCheck2 vector (Promega) downstream of the renilla luciferase gene. PsiCheck2 vector (Promega) was used as a control vector. TWIST-mut33-luciferase reporter was generated by site-directed mutagenesis with the following primers: 5′-TTTATTGAGGACCCATGGTAACATATGAATAGATCCGGTGTCTAAATGC-3′ (forward) and 5′-GCATTTAGACACCGGATCTATTCATATGTTACCATGGGTCCTCAATAAA-3′ (reverse). The miRNA-33 anti-seed sequence was converted to NdeI restriction site (underlined in the primers). Antagomir-33a (427064-00hsa-miR-33amiRCURY LNA™ microRNA Power inhibitor) was purchased from Exiqon (Woburn, MA, USA). miRNAs potentially able to suppress TWIST expression were selected by using TargetScan prediction software (http://www.targetscan.org). The miR-Vecs (miRNA expressing vectors) and MSCV-hTR (control vector) constructs were made as previously described [18].

### miRNA microarray analysis

Total RNA from OS tissues of the discovery cohort of patients was isolated using TRIzol reagent. The integrity of RNA was confirmed by agarose gel electrophoresis and its concentration determined by spectrophotometry. TaqMan Low Density miRNA Arrays (Applied Biosystems, Carlsbad, CA, USA) was used to assay the expression of human miRNAs by the manufacturer’s protocol. Manual inspection of all amplification plots was performed and miRNAs were excluded from the analysis if CT values were too high (>35, indicating that a miRNA expression is too low for accurate detection). Data analysis was performed using SDS 2.3 software (Applied Biosystems), which utilizes the delta-delta CT method [19].

### Real-time quantitative reverse transcription PCR

Total RNA was prepared from OS tissues or cell lines using TRIzol reagent followed by purification with TURBO DNA-free System (Ambion, Austin, TX). The cDNAs were synthesized using SuperScript II reverse transcriptase (Invitrogen, Carlsbad, CA, USA). Real-time quantitative PCR was performed using SYBR Green PCR master mix (Applied Biosystems) in a 7300 Real-time PCR System (Applied Biosystems). TaqMan microRNA assays (Applied Biosystems) that include RT primers and TaqMan probes were used to quantify the expression of mature miRNA-33a. The mean Ct was determined from triplicate PCRs. Gene expression was calculated relative to GAPDH. For measurement of TWIST mRNA, the following primers were used: for human *TWIST,* 5′-ACGAGCTGGACTCCAAGATG-3′ (forward) and 5′-CACGCCCTGTTTCTTTGAAT-3′ (reverse); for human *GAPDH*, 5′-GACTCATGACCACAGTCCATGC-3′ (forward) and 5′-AGAGGCAGGGATGATGTTCTG-3′ (reverse). The results were normalized against that of the *GAPDH* gene in the same sample. Each experiment was repeated for two times in triplicates.

### Western blot analysis

Briefly, cells were dissolved in 250 μl of 2× SDS loading buffer (62.5 mM TrisHCl, pH 6.8, 2% SDS, 25% glycerol, 0.01% bromphenol blue, 5% 2-mercaptoethanol), and incubated at 95°C for 10 min. Equal amount of proteins for each sample were separated by 10% SDS-polyacrylamide gel and blotted onto a polyvinylidene difluoride microporous membrane (Millipore, Billerica, MA, USA). Membranes were incubated for 1 h with a 1/1000 dilution of primary antibody, and then washed and revealed using secondary antibodies with horseradish peroxidase conjugate (1/5000, 1 h). Peroxidase was revealed with a GE Healthcare ECL kit (Shanghai, China).

### Transfection and lentiviral transduction

Plasmid constructs were transfected into cells using Superfect™ transfection reagent (Qiagen) according to the manufacture’s instructions. Pools of stable transfectants of TWIST were generated via selection with G418 (800 μg/ml) by the manufacturer’s protocol. Lentiviral transduction of TWIST-shRNA was performed and pools of stable transductants were generated via selection with puromycin (5 μg/ml).

### Luciferase assay

MG-63 cells were transfected with luciferase reporter constructs using Superfect™ transfection reagent (Qiagen). Luciferase activity was measured 72 hours after transfection using the Dual-luciferase reporter assay system (Promega) following the manufacturer’s instructions. Experiments were conducted in triplicates and results were expressed as ratios between renilla and firefly luciferase counts.

### Measurement of apoptosis by TUNEL (terminal deoxynucleotidyl transferase mediated nick-end labeling) assay

The TUNEL assay was performed using the DeadEnd™ Fluorometric TUNEL System by the manufacturer’s protocol (Promega). Cells were treated with cisplatin (15 nM) for 8 hours. Apoptotic cells exhibit a strong nuclear green fluorescence that could be detected using a standard fluorescein filter. All cells stained with DAPI exhibit a strong blue nuclear fluorescence. The slides were observed under fluorescence microscopy with relative apoptotic cells determined by counting TUNEL-positive cells in five random fields (magnification, ×100) for each sample.

### Statistical analysis

Statistical analyses were performed with SPSS for Windows 10.0. All continuous variable values were expressed as Mean±SD. Comparison of means between two groups was performed with student t tests. Comparisons of means among multiple groups were performed with one-way ANOVA followed by *post hoc* pairwise comparisons using Tukey’s tests. A two-tailed *p* < 0.05 was considered statistically significant in this study.

## Results

### miRNA expression profiling in chemoresistant and control OS

In the discovery cohort, patients were matched by age, sex and tumor stage. Thus, there was no significant difference in age, sex and distribution of tumor stages between poor responders (chemoresistant OS patients, n = 6, tumor necrosis 70.8% ± 9.2%) and good responders (control OS patients, n = 6, tumor necrosis 94.1% ± 2.8%) to neoadjuvant chemotherapy (Table 1). All patients had OS in the long tubular bones. As the inclusion rate for adult OS patients was low, we only performed this study with pediatric OS patients. miRNA microarray analyses showed that 25 miRNAs were differentially expressed in OS tissues from the chemoresistant OS patients compared with those from the control OS patients, 16 being up-regulated (Table 2) and 9 down-regulated (Table 3).

**Table 2:** Up-regulated miRNAs in chemoresistant vs control osteosarcomas

**miRNA**	**Ct (chemoresistant)**	**Ct (control)**	**∆∆ct**
hsa-miR-132	22.81	29.40	-8.67
hsa-miR-21	25.16	32.13	-7.54
hsa-miR-33a	21.57	28.70	-7.40
hsa-miR-215	26.97	33.13	-6.93
hsa-miR-221	26.15	32.07	-6.90
hsa-miR-140	27.62	32.78	-5.94
hsa-miR-375	22.46	26.92	-4.66
hsa-miR-181a	23.22	28.09	-3.96
hsa-miR-30a	22.62	25.75	-3.53
hsa-miR-25	31.54	33.87	-2.97
hsa-miR-200c	31.96	32.86	-2.61
hsa-miR-26b	26.70	29.17	-2.50
hsa-miR-23a	30.05	33.08	-2.42
hsa-miR-625	29.36	31.12	-2.11
hsa-miR-363	25.73	28.24	-2.09
hsa-miR-17-5p	31.02	32.25	-2.05

**Table 3:** Down-regulated miRNAs in chemoresistant vs control osteosarcomas

**miRNA**	**Ct (chemoresistant)**	**Ct (control)**	**∆∆ct**
hsa-miR-451	33.46	29.79	3.21
hsa-miR-92a	33.29	29.14	4.25
hsa-miR-200a	32.85	28.31	4.54
hsa-miR-15b	32.08	27.15	4.93
hsa-miR-143	31.15	24.85	5.27
hsa-miR-145	30.37	23.66	5.45
hsa-miR-422a	30.15	22.74	6.32
hsa-miR-611	29.72	22.96	6.76
hsa-miR-34c	28.64	21.29	7.35

### Screening of miRNAs able to regulate TWIST

**Figure 1:**
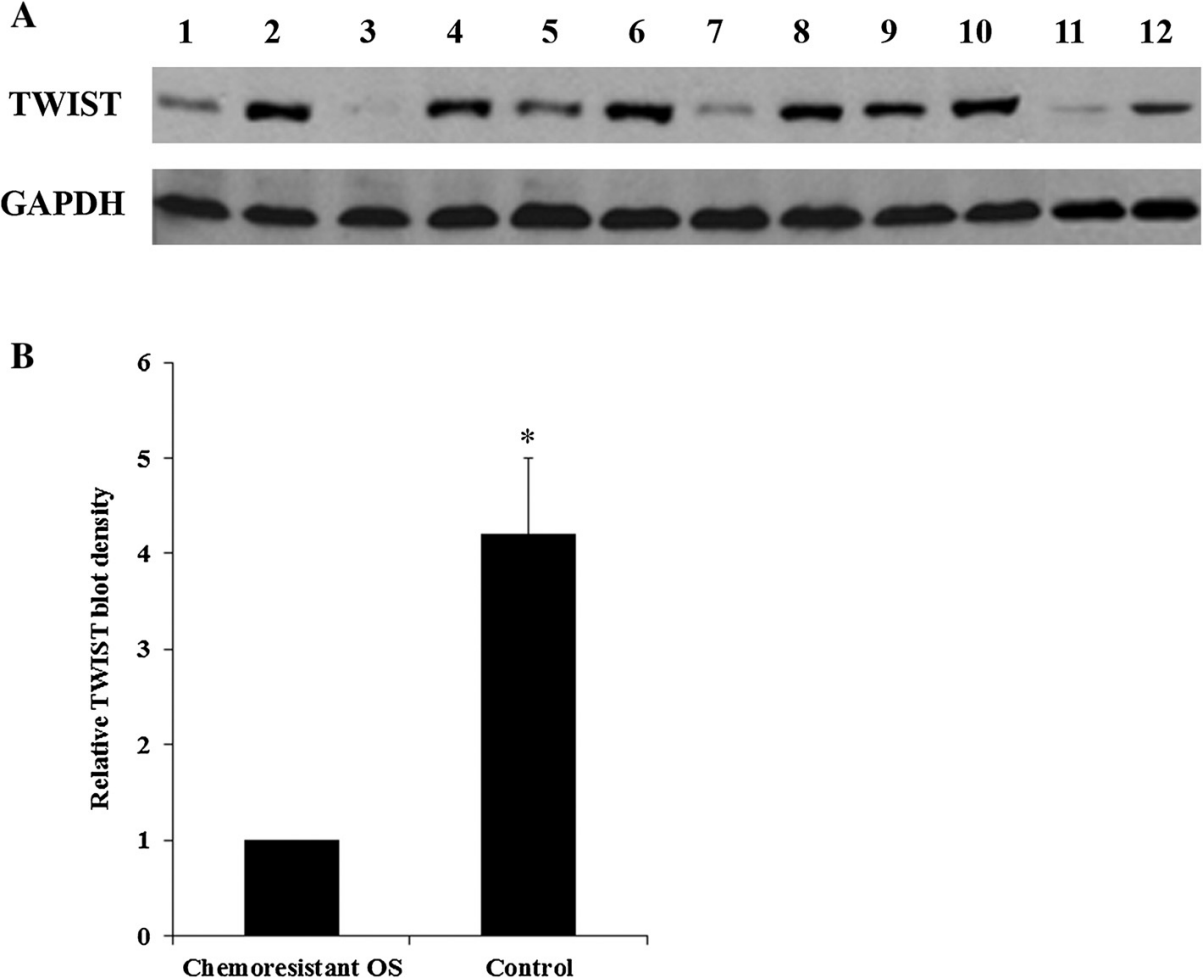
**Western blot analysis of TWIST expression in chemoresistant and control osteosarcoma (OS) tissues. (A)** OS tissue lysates from the chemoresistant OS and the non-chemoresistant control groups (n = 6 each) were subject to western blot analysis for TWIST expression. Glyceraldehyde-3-phosphate dehydrogenase (GAPDH*)* blotting was used as a loading control. Lanes 1, 3, 5, 7, 9, 11 were samples from the chemoresistant OS group. Lanes 2, 4, 6, 8, 10, 12 were samples from the control group. **(B)** Density of the TWIST blots was normalized against that of GAPDH to obtain a relative blot density. The relative TWIST blot density of the non-chemoresistant control group was expressed as fold changes to that of the chemoresistant OS group (designated as 1). *p < 0.05 vs chemoresistant OS.

**Figure 2:**
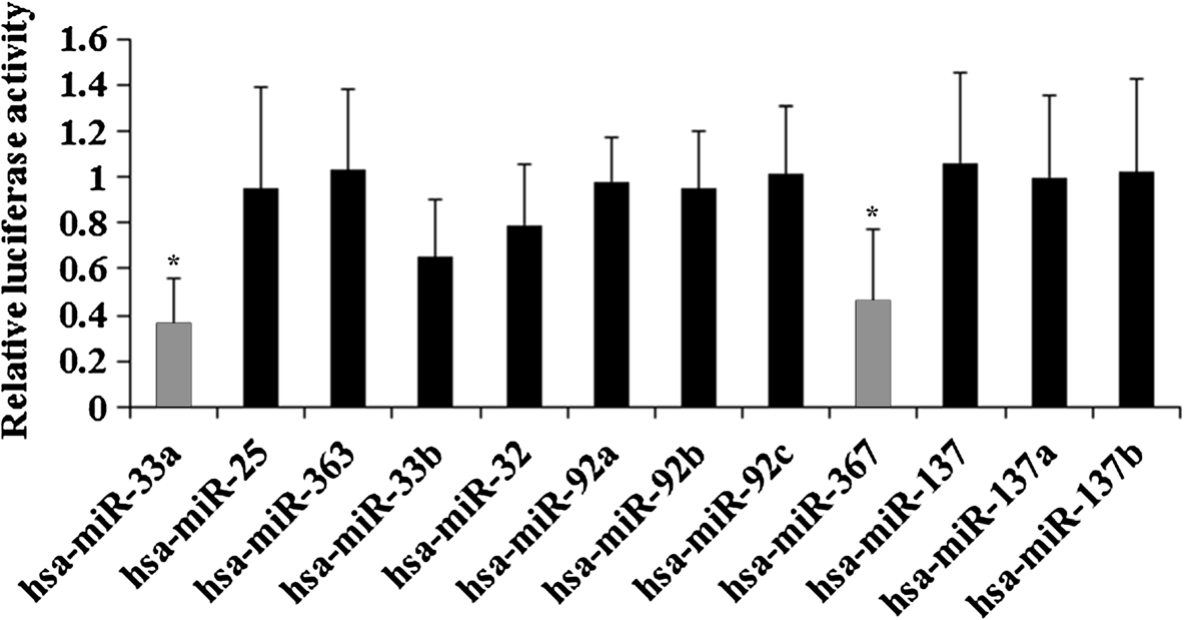
**Effects of selected miRNAs on TWIST 3′-untranslated region (UTR).** Twelve miRNAs potentially able to regulate TWIST 3′-UTR were selected based on TargetScan prediction software and individually co-transfected with a TWIST-3′UTR-luciferase reporter in MG-63 cells. Among the selected miRNAs, miR-33a, miR-25 and miR-363 were differentially expressed between chemoresistant and control osteosarcoma tissues based on results of the microarray analysis. miRNAs that significantly reduced renilla luciferase activity compared to the control miR-Vec (hTR) (designated as 1, cut off value: 0.8) were selected for validation (light bars). *p < 0.05 vs 0.8 (cut off value).

**Figure 3:**
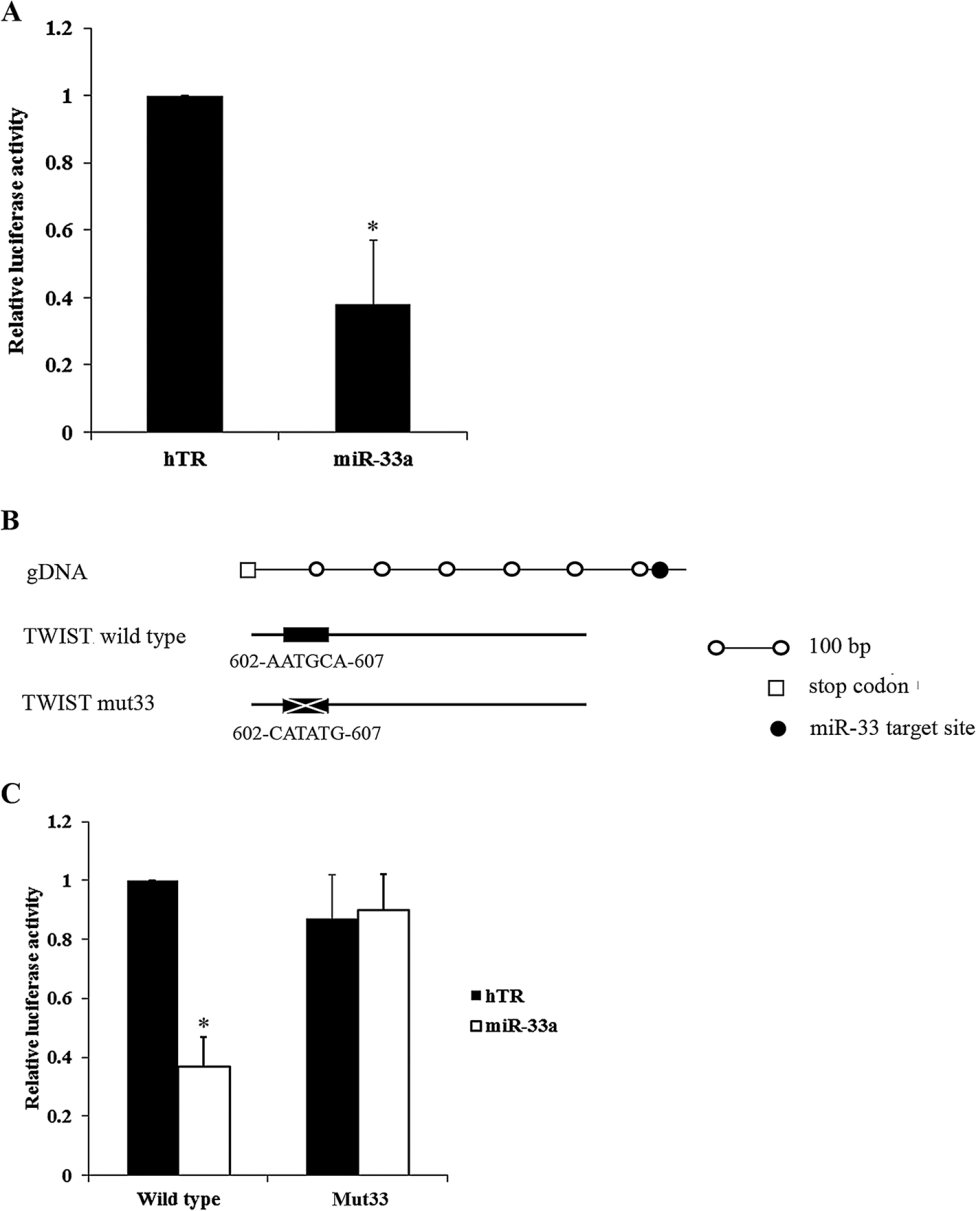
**Regulation of TWIST 3′-untranslated region (UTR) by miR-33a. (A)** MG-63 cells were co-transfected with a TWIST-3′UTR-luciferase reporter and miR-33a or control miR-Vec (hTR). The relative luciferase activity in cells co-transfected with hTR was designated as 1. **(B)** Schematic presentation of generation of a TWIST-mut33-luciferase reporter (mut33) by site-directed mutagenesis of the predicted binding sequence of miR-33a in TWIST 3′-UTR. **(C)** MG-63 cells were co-transfected with the TWIST-3′UTR-luciferase reporter or TWIST-mut33-luciferase reporter and miR-33a or control miR-Vec (hTR). The relative luciferase activity in cells co-transfected with the TWIST-3′UTR-luciferase reporter (wild type) and hTR was designated as 1. *p < 0.05 vs hTR.

The expression of TWIST in the chemoresistant OS patients was significantly lower than that in the control OS patients, either individually or by group (Figure 1), suggesting that TWIST is an important negative regulator in the development of OS chemoresistance. We screened for miRNAs able to regulate TWIST in OS. To this purpose, the 3′-UTR of the TWIST gene was inserted downstream of the renilla luciferase gene in the psiCheck2 vector to generate a TWIST-3′UTR-luciferase reporter. By combining the microarray expression data with results of the prediction software TargetScan, three up-regulated miRNAs (miR-33a, miR-25 and miR-363) potentially able to regulate TWIST 3′-UTR were selected and individually tested for their ability to affect luciferase expression in MG-63 human OS cells co-transfected with the TWIST-3′UTR-luciferase reporter. In addition, a group of nine miRNAs (miR-33b, miR-32, miR-92a, miR-92b, miR-92c, miR-367, miR-137, miR-137a, and miR-137b) predicted by TargetScan but not differentially expressed in our microarray analysis were also assayed (Figure 2). miR-Vec control (hTR) was used as a negative control. We used MG-63 cells because, according to our microarray data, the predicted miRNAs were absent or expressed at very low levels in the cell line (data not shown). miR-33a, miR-33b and miR-367 significantly reduced renilla luciferase activity compared to the control miR-Vec (cut off value: 0.8) (Figure 2), and miR-33a was found the best candidate to significantly and consistently reduce renilla luciferase activity compared to the control (Figure 3*A*).

To demonstrate a direct interaction between miR-33a and TWIST, the potential binding sequence for the miRNA within the 3′-UTR of TWIST, as predicted by TargetScan, was mutated to generate a TWIST-mut33-luciferase reporter (Figure 3*B*). MG-63 cells were co-transfected with miR-33a or miR-Vec control together with either TWIST-3′UTR-luciferase reporter or TWIST-mut33-luciferase reporter. The reduction of renilla luciferase activity caused by miRNA-33a was specifically abolished by the mutation of the corresponding anti-seed sequence (Figure 3*C*), suggesting that miR-33a could suppress TWIST expression by acting on its predicted sequence in the 3′-UTR.

**Figure 4:**
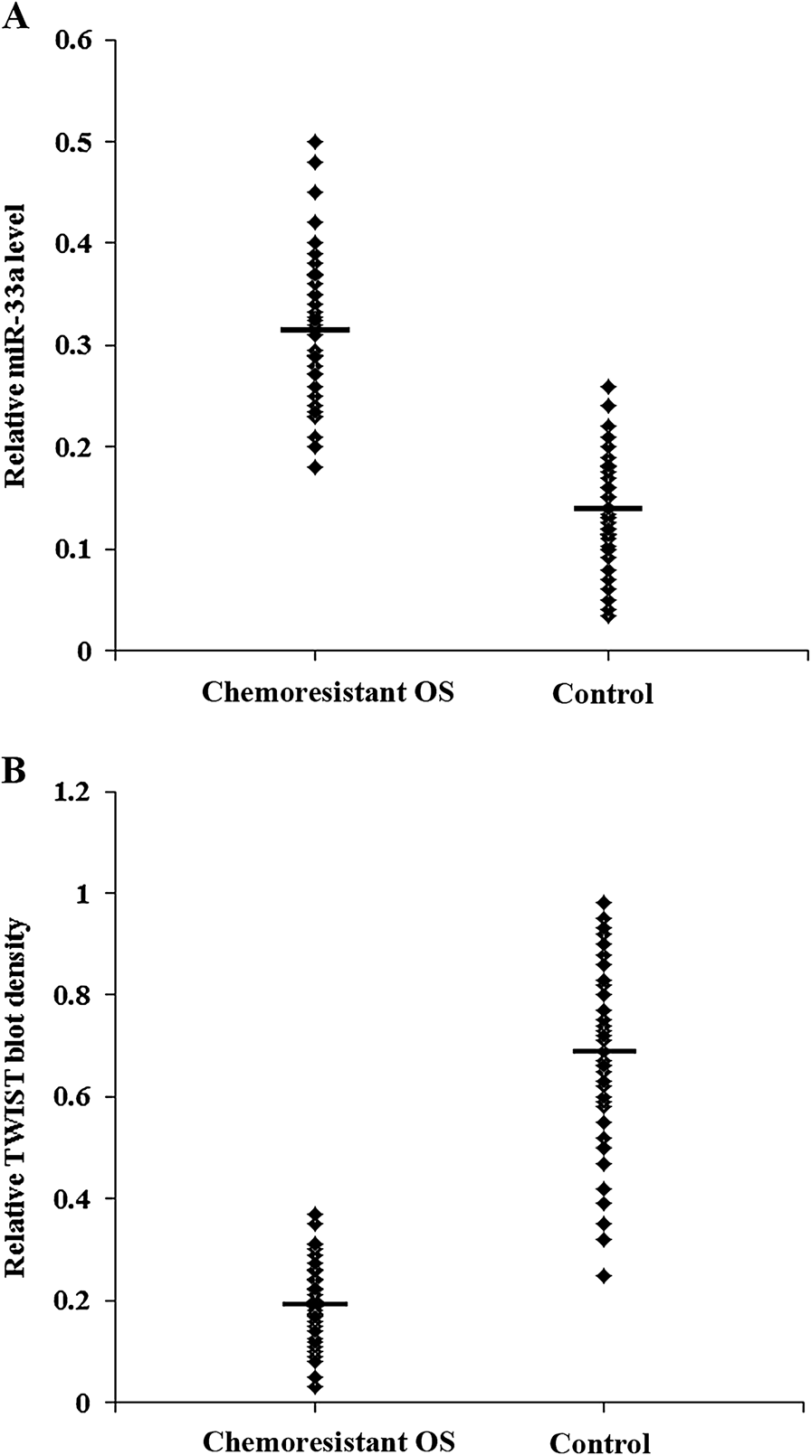
**miR-33a and TWIST levels in osteosarcoma (OS) tissues from chemoresistant and control OS patients in the validation cohort.** Real-time RT-PCR and Western blot analysis were performed to determine **(A)** miR-33a and **(B)** TWIST protein levels in OS tissues from the chemoresistant OS and control groups in the validation cohort (n = 35 each group), respectively. Data are shown in scatter plots. The mean miR-33a and TWIST protein levels are marked by a horizontal bar in each group.

To confirm the findings, we determined miRNA-33a and TWIST protein levels in chemoresistant OS patients (n = 35) and control patients (n = 35) in the validation cohort. As shown in Figure 4*A*, the chemoresistant OS group presented a significantly higher range of miR-33a levels than the control group (0.32 ± 0.08 vs 0.13 ± 0.05; *p* < 0.001). On the other hand, the chemoresistant OS group presented a significantly lower range of TWIST protein levels than the control group (0.20 ± 0.08 vs 0.67 ± 0.19; *p* < 0.001) (Figure 4*B*). Correlation analyses in the entire validation cohort (n = 70) showed that the miR-33a level was negatively correlated with the TWIST protein level in the OS tissue (r = -0.627, *p* < 0.001). The miR-33a was negatively correlated with the tumor necrosis rate (r = -0.352, *p* < 0.001), while the TWIST protein level was positively correlated with the tumor necrosis rate (r = 0.562, *p* < 0.001).

### Effect of overexpression and inhibition of miR-33a on TWIST expression in OS cells

**Figure 5:**
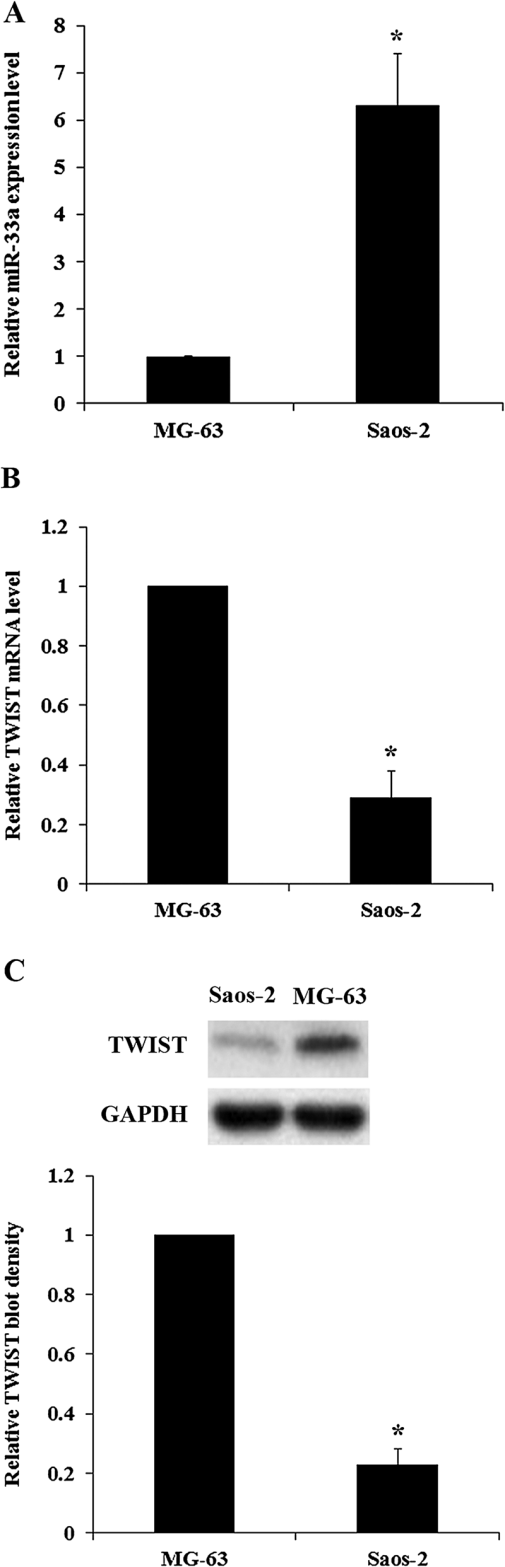
**miR-33a and TWIST levels in osteosarcoma (OS) cells.** Real-time RT-PCR and Western blot analysis were performed to determine **(A)** miR-33a and **(B)** TWIST mRNA and **(C)** TWIST protein levels in Saos-2 and MG-63 human OS cells. The miR-33a expression level and the relative TWIST mRNA level and protein blot density in MG-63 cells were designated as 1, respectively. *p < 0.05 vs MG-63.

**Figure 6:**
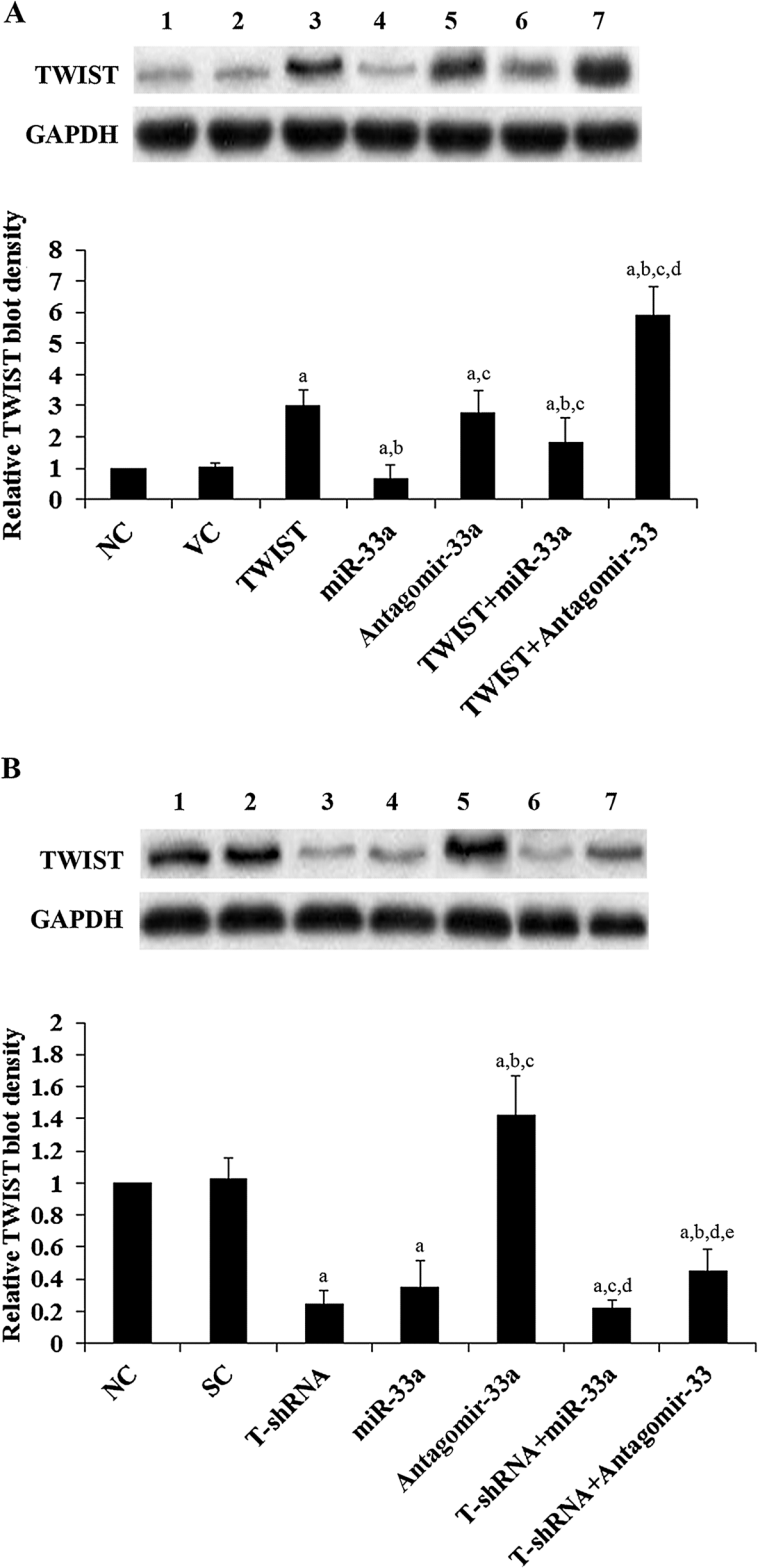
**TWIST expression in osteosarcoma cells with overexpression or knockdown/inhibition of TWIST and/or miR-33a. (A)** In Saos-2 cells, TWIST expression was determined in normal control cells (NC, lane 1), cells stably transfected with empty pcDNA3 vector (VC, lane 2), cells overexpressing TWIST (lane 3), cells overexpressing miR-33a (lane 4), cells transfected with antagomir-33a (lane 5), cells overexpressing TWIST and miR-33a (lane 6), and cells overexpressing TWIST plus transfection of antagomir-33a (lane 7). **(B)** In MG-63 cells, TWIST expression was determined in normal control cells (NC, lane 1), cells stably transduced with scramble control shRNA (SC, lane 2), cells stably expressing TWIST-shRNA (T-shRNA, lane 3), cells overexpressing miR-33a (lane 4), cells transfected with antagomir-33a (lane 5), cells stably expressing T-shRNA and overexpressing miR-33a (lane 6), and cells stably expressing T-shRNA plus transfection of antagomir-33a (lane 7). Glyceraldehyde-3-phosphate dehydrogenase (GAPDH*)* blotting was used as a loading control. Density of the TWIST blot was normalized against that of GAPDH to obtain a relative blot density, which was expressed as fold changes to the relative TWIST blot density of NC cells (designated as 1). In **(A)** Saos-2 cells, ^a^*p* < 0.05 vs NC and VC; ^b^*p* < 0.05 vs TWIST; ^c^*p* < 0.05 vs miR-33a; ^d^*p* < 0.05 vs antagomir-33a. In **(B)** MG-63 cells, ^a^*p* < 0.05 vs NC and SC; ^b^*p* < 0.05 vs T-shRNA; ^c^*p* < 0.05 vs miR-33a; ^d^*p* < 0.05 vs antagomir-33a; ^e^*p* < 0.05 vs T-shRNA + miR-33a.

We next examined the effects of miRNA-33a on TWIST expression in human OS cells. As shown in Figure 5, miR-33a was highly expressed in Saos-2 cells, which had a low constitutive expression of TWIST at both the mRNA and the protein levels. In contrast, MG-63 cells had a constitutive low expression of miR-33a, and a high expression of TWIST at both the mRNA and the protein levels (Figure 5). Thus, overexpression and knockdown of TWIST were respectively performed in the two cell lines to approach the study objectives. As shown in Figure 6*A*, inhibition of miR-33a by antagomir-33a increased TWIST expression by over 1.5 fold in Saos-2 cells. On the other hand, overexpression of miR-33a decreased TWIST expression by about 30%. Overexpression of TWIST led to an approximately two-fold increase of TWIST expression in Saos-2 cells, which was largely reversed by overexpression of miR-33a and doubled by antagomir-33a. As shown in Figure 6*B*, overexpression of miR-33a decreased TWIST expression by nearly 70% in MG-63 cells, while antagomir-33a increased TWIST expression by 0.4 fold. Knockdown of TWIST by shRNA resulted in an approximately 80% decrease of endogenous TWIST expression in MG-63 cells, which was partially reversed by antagomir-33a.

### Functional role of miR-33a in TWIST-inhibited OS cell survival against cisplatin

**Figure 7:**
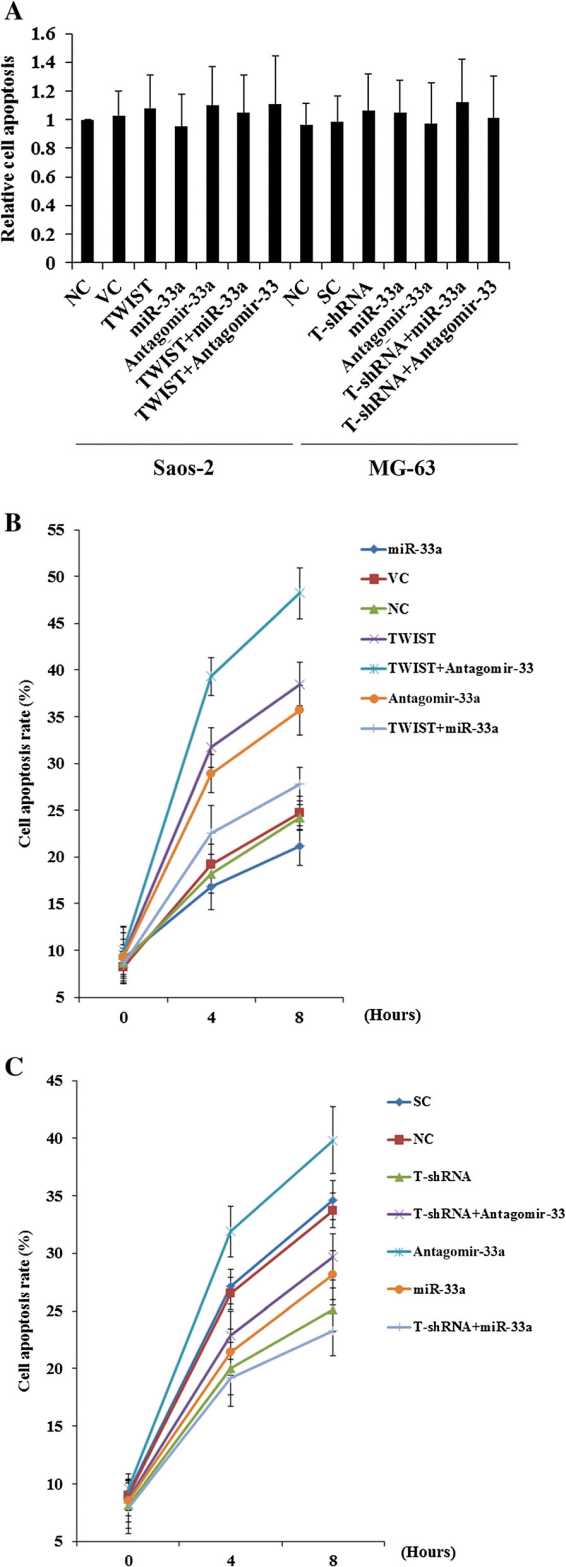
**Cisplatin-induced apoptosis in osteosarcoma cells with overexpression or knockdown/inhibition of TWIST and/or miR-33a.** In Saos-2 cells, TUNEL (terminal deoxynucleotidyl transferase mediated nick-end labeling) assays were performed in normal control cells (NC), cells stably transfected with empty pcDNA3 vector (VC), cells overexpressing TWIST, cells overexpressing miR-33a, cells transfected with antagomir-33a, cells overexpressing TWIST and miR-33a, and cells overexpressing TWIST plus transfection of antagomir-33a. In MG-63 cells, TUNEL assays were performed in normal control cells (NC), cells stably transduced with scramble control shRNA (SC), cells stably expressing TWIST-shRNA (T-shRNA), cells overexpressing miR-33a, cells transfected with antagomir-33a, cells stably expressing T-shRNA and overexpressing miR-33a, and cells stably expressing T-shRNA plus transfection of antagomir-33a. **(A)** The cells were under normal culture conditions for 8 hours. The cell apoptosis rate at 8 hours was expressed as fold changes to that of the Saos-2 NC cells (designated as 1). **(B)** Saos-2 and **(C)** MG-63 cells were treated with 15 nM of cisplatin for 8 hours. The cell apoptosis rates at 4 hours and 8 hours were shown as the percentage of TUNEL positive cells in total cells.

TWIST reportedly decreases OS cell survival against cisplatin, an apoptosis-inducing chemotherapeutic agent commonly used to treat OS [10,11,16]. To explore the effect of interaction between miR-33a and TWIST on OS chemoresistance, we examined cell apoptosis rate in both cell lines treated with cisplatin (15 nM) using TUNEL (terminal deoxynucleotidyl transferase mediated nick-end labeling) assays. Overexpression or knockdown/inhibition of TWIST and/or miR-33a did not significantly alter cell apoptosis in both Saos-2 and MG-63 cells under normal culture conditions (Figure 7*A*). In Saos-2 cells treated with cisplatin, inhibition of miR-33a by antagomir-33a markedly increased cell apoptosis, which was enhanced by overexpression of TWIST (Figure 7*B*). The apoptosis-inducing effect of TWIST overexpression was reversed by overexpression of miR-33a (Figure 7*B*). In MG-63 cells, overexpression of miR-33a significantly decreased cisplatin-induced cell apoptosis, which was enhanced by knockdown of TWIST (Figure 7*C*). Antagomir-33a significantly increased cisplatin-induced cell apoptosis, which was reversed by knockdown of TWIST (Figure 7*C*).

## Discussion

Chemoresistance is the major reason for poor survival of OS patients. Previous studies reported that TWIST could decrease OS cell survival against cisplatin by inhibiting multiple signaling pathways [10,11], suggesting that TWIST is a pivotal negative regulator of OS chemoresistance. miRNAs reportedly are involved in the pathogenesis and chemoresistance of various cancers, including OS. In the present study, we profiled miRNAs differentially expressed in chemoresistant OS by microarray analysis, with a focus to identify miRNAs that regulate TWIST expression and OS chemoresistance. We provide the first evidence suggesting that miR-33a promotes OS chemoresistance by down-regulating TWIST.

OS is the most common pediatric bone malignancy in the world [1]. As the inclusion rate for adult OS patients was low, we performed this study only in pediatric OS patients. Patients (n = 12) in the discovery cohort were matched on age, sex and tumor stages to reduce the effects of confounders on miRNA profiling between chemoresistant and control OS samples. Patients (n = 70) in the validation cohort were not matched in order to verify the profiling findings in a more generalizable setting. Among the up-regulated miRNAs identified in chemoresistant OS samples in this study, miR-140, miR-215 and miR-221 have been reported to induce human OS chemoresistance [20-22]. Among the down-regulated miRNAs identified in chemoresistant OS samples, miR-451 and miR-15b have been reported to increase chemosensitivity of OS [13]. Thus, our findings were in agreement with previous studies, indicating good reliability of the data.

High expression of TWIST has been detected in several cancers and has been associated with the initial phase of metastatic progression [23]. One recent study reported that TWIST overexpression correlated with disease progression and a poor clinical outcome in OS patients [23]. On the other hand, it has been reported that in homogeneous cohort of OS patients, the *TWIST* gene was frequently deleted in the tumors at diagnosis, and its haploinsufficiency was significantly correlated with a poorer patient outcome [6,9]. In addition, two recent studies reported that TWIST could decrease OS cell survival against cisplatin by inhibiting β-catenin signaling and endothelin-1/endothelin A receptor signaling pathways [10,11], suggesting that TWIST is an important negative regulator in the development of OS chemoresistance. In this study, our in vitro results showed that overexpression and knockdown of TWIST increased and decreased cisplatin-induced OS cell apoptosis, respectively. This was corroborated by our findings that the expression of TWIST in the chemoresistant OS group was significantly lower than that in the control OS group in both the discovery and validation cohorts, which provides further evidence supporting a critical counteracting role of TWIST in the development of OS chemoresistance.

With an aim to identify miRNAs regulating TWIST expression in OS, we found that miR-33a could significantly down-regulate TWIST expression, which was supported by an inverse miRNA-33a/TWIST expression trend in the validation cohort, target-sequence-specific inhibition of TWIST-3′UTR-luciferase reporter activity by miR-33a, and alteration of TWIST expression by overexpression or inhibition of miR-33a in human OS cell lines. Saos-2 and MG-63 cells were employed as OS cell models in this study. Saos-2 cells have a constitutive high expression of miR-33a and low expression of TWIST, while MG-63 cells have a constitutive low expression of miR-33a and high expression of TWIST. This explains why inhibition of miR-33a by antagomir-33a had more pronounced effects on TWIST expression than overexpressing miR-33a in Saos-2 cells. Likewise, overexpressing miR-33a had more pronounced effects on TWIST expression than antagomir-33a treatment in MG-63 cells. The effects of overexpression and inhibition of miR-33a on TWIST expression significantly altered OS cell resistance to cisplatin, a chemotherapeutic agent routinely used in neoadjuvant chemotherapy for OS [16]. In the presence of cisplatin, antagomir-33a significantly enhanced cisplatin-induced apoptosis in both Saos-2 and MG-63 cells, suggesting that inhibition of miR-33a could be a potential new strategy to enhance neoadjuvant chemotherapy for OS. The effects of antagomir-33a was reversed and enhanced by knockdown and overexpression of TWIST, respectively, indicating that miR-33a promotes OS cell resistance to cisplatin by down-regulating TWIST, or antagomir-33a enhances cisplatin-induced OS cell apoptosis by up-regulating TWIST. miR-33a has been shown to regulate genes involved in fatty acid metabolism and insulin signaling [24]. A recent study indicated that miR-33a targets the proto-oncogene Pim-1 and suggested overexpression of miR-33a as an anticancer treatment [25]. However, another study described the down-regulation of tumor suppressor p53 by miR-33 [26], suggesting a complex and possible context-dependent response to miR-33 manipulations. As p53 is often mutated in OS [27], it is unlikely that miR-33a promotes OS chemoresistance through down-regulating p53-induced apoptosis. Thus, the enhancing effect of miR-33a on OS chemoresistance via down-regulating TWIST expression is a new function of this miR, and the miR-33a/TWIST signaling could be a novel mechanism involved in development of OS chemoresistance.

There are some limitations of this study: [1] This study was only performed in pediatric OS patients. Despite that adult OS patients only occupy a small portion of total OS patients, it would still be interesting to verify the findings in adult patients in future studies. [2] Cisplatin elicits DNA repair mechanisms by crosslinking DNA, which in turn activates apoptosis when repair proves impossible [28]. In this study, we only examined the effect of miR-33a/TWIST signaling on OS cell resistance to cisplatin. It is unclear whether miR-33a/TWIST would impact OS cell resistance to other types of chemotherapy agents. Further studies with more types of chemotherapy agents and OS cell lines would elaborate this issue.

In conclusion, we demonstrate that miR-33a is up-regulated in chemoresistant OS and that the miR-33a level is negatively correlated with the TWIST protein level and the tumor necrosis rate in OS. Our in vitro data indicate that miR-33a promotes OS cell resistance to cisplatin by down-regulating TWIST; on the other hand, inhibition of miR-33a by antagomir-33a enhances cisplatin-induced apoptosis in OS cells by up-regulating TWIST expression. The findings suggest that inhibition of miR-33a/TWIST signaling could be a potential new strategy to enhance neoadjuvant chemotherapy for OS.

## Competing interests

The authors declare that they have no competing interests.

## Authors’ contributions

YZ participated in study design, collected data, carried out data analysis, and drafted the manuscript. SW and XZ participated in study design, carried out data analysis, and performed data check and proofreading. ML and JS participated in data collection, carried out data analysis, and performed data check and proofreading. ZH participated in study design and data analysis, drafted the manuscript, and performed data check and proofreading. All authors have read and approved the final manuscript.

## References

[B1] Ottaviani G Jaffe N Jaffe N The epidemiology of osteosarcoma Pediatric and Adolescent Osteosarcoma 2009 New York: Springer 122 136

[B2] Subbiah V Kurzrock R Phase 1 clinical trials for sarcomas: the cutting edge Curr Opin Oncol 2011 23 352 360 10.1097/CCO.0b013e3283477a94 21519259

[B3] Chou AJ Gorlick R Chemotherapy resistance in osteosarcoma: current challenges and future directions Expert Rev Anticancer Ther 2006 6 1075 1085 10.1586/14737140.6.7.1075 16831079

[B4] Uribe-Botero G Russell WO Sutow WW Martin RG Primary osteosarcoma of bone. Clinicopathologic investigation of 243 cases, with necropsy studies in 54 Am J Clin Pathol 1977 67 427 435 266360 10.1093/ajcp/67.5.427

[B5] Geller DS Gorlick R Osteosarcoma: a review of diagnosis, management, and treatment strategies Clin Adv Hematol Oncol 2010 8 705 718 21317869

[B6] Entz-Werlé N Lavaux T Metzger N Stoetzel C Lasthaus C Marec P Kalifa C Brugieres L Pacquement H Schmitt C Tabone MD Gentet JC Lutz P Babin A Oudet P Gaub MP Perrin-Schmitt F Involvement of MET/TWIST/APC combination or the potential role of ossification factors in pediatric high-grade osteosarcoma oncogenesis Neoplasia 2007 9 678 688 10.1593/neo.07367 17786187 PMC1950438

[B7] Stoetzel C Weber B Bourgeois P Bolcato-Bellemin AL Perrin-Schmitt F Dorso-ventral and rostro-caudal sequential expression of M-twist in the postimplantation murine embryo Mech Dev 1995 51 251 263 10.1016/0925-4773(95)00369-X 7547472

[B8] El Ghouzzi V Le Merrer M Perrin-Schmitt F Lajeunie E Benit P Renier D Bourgeois P Bolcato-Bellemin AL Munnich A Bonaventure J Mutations of the TWIST gene in the Saethre-Chotzen syndrome Nat Genet 1997 15 42 46 10.1038/ng0197-42 8988167

[B9] Le Deley MC Guinebretière J Gentet JC Pacquement H Pichon F Marec-Bérard P Entz-Werlé N Schmitt C Brugières L Vanel D Dupoüy N Tabone MD Kalifa C SFOP OS94: a randomised trial comparing preoperative high-dose methotrexate plus doxorubicin to high-dose methotrexate plus etoposide and ifosfamide in osteosarcoma patients Eur J Cancer 2007 43 752 761 10.1016/j.ejca.2006.10.023 17267204

[B10] Wu J Liao Q He H Zhong D Yin K TWIST interacts with β-catenin signaling on osteosarcoma cell survival against cisplatin Mol Carcinog 2012 10.1002/mc.21991 10.1002/mc.21991 23280703

[B11] Zhou Y Zang X Huang Z Zhang C TWIST interacts with endothelin-1/endothelin A receptor signaling in osteosarcoma cell survival against cisplatin Oncol Lett 2013 5 857 861 23426781 10.3892/ol.2013.1111 PMC3576190

[B12] Ma R Jiang T Kang X Circulating microRNAs in cancer: origin, function and application J Exp Clin Cancer Res 2012 31 38 10.1186/1756-9966-31-38 22546315 PMC3431991

[B13] Jones KB Salah Z Del Mare S Galasso M Gaudio E Nuovo GJ Lovat F LeBlanc K Palatini J Randall RL Volinia S Stein GS Croce CM Lian JB Aqeilan RI miRNA signatures associate with pathogenesis and progression of osteosarcoma Cancer Res 2012 72 1865 1877 10.1158/0008-5472.CAN-11-2663 22350417 PMC3328547

[B14] Liu X Chen X Yu X Tao Y Bode AM Dong Z Cao Y Regulation of microRNAs by epigenetics and their interplay involved in cancer J Exp Clin Cancer Res 2013 32 96 10.1186/1756-9966-32-96 24261995 PMC3874662

[B15] Nana-Sinkam SP Croce CM MicroRNAs as therapeutic targets in cancer Transl Res 2011 157 216 225 10.1016/j.trsl.2011.01.013 21420032

[B16] Bacci G Bertoni F Longhi A Ferrari S Forni C Biagini R Bacchini P Donati D Manfrini M Bernini G Lari S Neoadjuvant chemotherapy for high-grade central osteosarcoma of the extremity. Histologic response to preoperative chemotherapy correlates with histologic subtype of the tumor Cancer 2003 97 3068 3075 10.1002/cncr.11456 12784343

[B17] Matsuo N Shiraha H Fujik T Takaoka N Ueda N Tanaka S Nishina S Nakanishi Y Uemura M Takaki A Nakamura S Kobayashi Y Nouso K Yagi T Yamamoto K Twist expression promotes migration and invasion in hepatocellular carcinoma BMC Cancer 2009 9 240 10.1186/1471-2407-9-240 19615090 PMC2720986

[B18] Voorhoeve PM Le Sage C Schrier M Gillis AJ Stoop H Nagel R Liu YP Van Duijse J Drost J Griekspoor A Zlotorynski E Yabuta N De Vita G Nojima H Looijenga LH Agami R A genetic screen implicates miRNA-372 and miRNA-373 as oncogenes in testicular germ cell tumors Cell 2006 124 1169 1181 10.1016/j.cell.2006.02.037 16564011

[B19] Schmittgen TD Livak KJ Analyzing real-time PCR data by the comparative C (T) method Nat Protoc 2008 3 1101 1108 10.1038/nprot.2008.73 18546601

[B20] Song B Wang Y Xi Y Kudo K Bruheim S Botchkina GI Gavin E Wan Y Formentini A Kornmann M Fodstad O Ju J Mechanism of chemoresistance mediated by miR-140 in human osteosarcoma and colon cancer cells Oncogene 2009 28 4065 4074 10.1038/onc.2009.274 19734943 PMC2783211

[B21] Song B Wang Y Titmus MA Botchkina G Formentini A Kornmann M Ju J Molecular mechanism of chemoresistance by miR-215 in osteosarcoma and colon cancer cells Mol Cancer 2010 9 96 10.1186/1476-4598-9-96 20433742 PMC2881118

[B22] Zhao G Cai C Yang T Qiu X Liao B Li W Ji Z Zhao J Zhao H Guo M Ma Q Xiao C Fan Q Ma B MicroRNA-221 induces cell survival and cisplatin resistance through PI3K/Akt pathway in human osteosarcoma PLoS One 2013 8 e53906 10.1371/journal.pone.0053906 23372675 PMC3553141

[B23] Yin K Liao Q He H Zhong D Prognostic value of Twist and E-cadherin in patients with osteosarcoma Med Oncol 2012 29 3449 3455 10.1007/s12032-012-0317-6 22847601

[B24] Dávalos A Goedeke L Smibert P Ramírez CM Warrier NP Andreo U Cirera-Salinas D Rayner K Suresh U Pastor-Pareja JC Esplugues E Fisher EA Penalva LO Moore KJ Suárez Y Lai EC Fernández-Hernando C miR-33a/b contribute to the regulation of fatty acid metabolism and insulin signaling Proc Natl Acad Sci USA 2011 108 9232 9237 10.1073/pnas.1102281108 21576456 PMC3107310

[B25] Thomas M Lange-Grunweller K Weirauch U Gutsch D Aigner A Grunweller A Hartmann RK The protooncogene Pim-1 is a target of miR-33a Oncogene 2012 31 918 928 10.1038/onc.2011.278 21743487

[B26] Herrera-Merchan A Cerrato C Luengo G Dominguez O Piris MA Serrano M Gonzalez S miR-33-Mediated downregulation of p53 controls hematopoietic stem cell self-renewal Cell Cycle 2010 9 3277 3285 20703086 10.4161/cc.9.16.12598

[B27] Kanamori M Sano A Yasuda T Hori T Suzuki K Array-based comparative genomic hybridization for genomic-wide screening of DNA copy number alterations in aggressive bone tumors J Exp Clin Cancer Res 2012 31 100 10.1186/1756-9966-31-100 23199169 PMC3576288

[B28] Rosenberg B Vancamp L Trosko JE Mansour VH Platinum compounds: a new class of potent antitumour agents Nature 1969 222 385 386 10.1038/222385a0 5782119

